# Development of a Novel Weighted Ranking Method for Immunohistochemical Quantification of a Heterogeneously Expressed Protein in Gastro-Esophageal Cancers

**DOI:** 10.3390/cancers13061286

**Published:** 2021-03-13

**Authors:** Cathy E. Richards, Katherine M. Sheehan, Elaine W. Kay, Charlotta Hedner, David Borg, Joanna Fay, Anthony O’Grady, Arnold D. K. Hill, Karin Jirström, Ann M. Hopkins

**Affiliations:** 1 Department of Surgery, Royal College of Surgeons in Ireland, Beaumont Hospital, Dublin 9, Ireland; catherinerichards@rcsi.com (C.E.R.); adkhill@rcsi.com (A.D.K.H.); 2 Department of Pathology, Royal College of Surgeons in Ireland, Beaumont Hospital, Dublin 9, Ireland; ksheehan@rcsi.ie (K.M.S.); ekay@rcsi.ie (E.W.K.); joannafay@rcsi.ie (J.F.); togrady@rcsi.ie (A.O.); 3 Department of Clinical Sciences Lund, Division of Oncology and Therapeutic Pathology, Lund University, SE 221 85 Lund, Sweden; charlotta.hedner@med.lu.se (C.H.); david.borg@med.lu.se (D.B.); karin.jirstrom@med.lu.se (K.J.)

**Keywords:** JAM-A, HER2, gastro-esophageal cancer, intra-tumoral heterogeneity, tumor, cancer, immunohistochemistry, tissue microarray

## Abstract

**Simple Summary:**

High levels of the protein Junctional Adhesion Molecule-A (JAM-A) have been linked with aggressive disease in patients with several different cancers. However, its distribution is often non-uniform (heterogeneous) across tumors, and can be difficult to quantify. JAM-A has also been linked with high levels of HER2 (an important oncogene) in breast tumors, and the development of resistance to HER2-targeted drugs in those patients. Since gastro-esophageal (GE) cancers are often high in HER2 and have also been approved for HER2-targeted drugs, the aim of this study was to investigate if levels of JAM-A and HER2 are linked in GE cancer. JAM-A was expressed very heterogeneously across miniaturized tissue sections called tissue microarrays (TMAs) of GE cancer patients. In this model, therefore, there was no correlation between JAM-A and HER2 expression. However, when we used larger tissue sections and developed a scoring system to account for heterogeneity, a significant correlation between JAM-A and HER2 levels emerged. This work illustrates the importance of taking intra-tumor heterogeneity into account, particularly in an era when analysis of protein levels by this method is increasingly used to select patients for targeted cancer drugs.

**Abstract:**

High expression of Junctional Adhesion Molecule-A (JAM-A) has been linked with poor prognosis in several cancers, including breast cancers overexpressing the human epidermal growth factor receptor-2 (HER2). Furthermore, JAM-A expression has been linked with regulating that of HER2, and associated with the development of resistance to HER2-targeted therapies in breast cancer patients. The purpose of this study was to establish a potential relationship between JAM-A and HER2 in HER2-overexpressing gastro-esophageal (GE) cancers. Interrogation of gene expression datasets revealed that high JAM-A mRNA expression was associated with poorer survival in HER2-positive gastric cancer patients. However, high intra-tumoral heterogeneity of JAM-A protein expression was noted upon immunohistochemical scoring of a GE cancer tissue microarray (TMA), precluding a simple confirmation of any relationship between JAM-A and HER2 at protein level. However, in a test-set of 25 full-face GE cancer tissue sections, a novel weighted ranking system proved effective in capturing JAM-A intra-tumoral heterogeneity and confirming statistically significant correlations between JAM-A/HER2 expression. Given the growing importance of immunohistochemistry in stratifying cancer patients for the receipt of new targeted therapies, this may sound a cautionary note against over-relying on cancer TMAs in biomarker discovery studies of heterogeneously expressed proteins. It also highlights a timely need to develop validated mechanisms of capturing intra-tumoral heterogeneity to aid in future biomarker/therapeutic target development for the benefit of cancer patients.

## 1. Introduction

Junctional Adhesion Molecule-A (JAM-A) is an immunoglobulin superfamily protein located at epithelial and endothelial tight junctions which plays important physiological roles in cell polarity and migration [1,2,3]. In pathophysiological contexts, we and others have reported JAM-A to be overexpressed in breast [4,5,6,7] and other cancers including gastric, nasopharyngeal, lung, ovarian and brain [8,9,10,11]. However, the role of JAM-A in gastro-esophageal (GE) cancers is unclear, with contradictory reports surrounding the aberrant expression of JAM-A in this setting [12,13]. Whether this reflects inter- or intra-tumor heterogeneity is currently unclear. 

Similar to aggressive breast cancers overexpressing the human epidermal growth factor receptor-2 (HER2); a proportion of GE cancers overexpress HER2 and have been approved for the use of HER2-targeting therapies in patients [14,15,16]. In light of evidence that JAM-A may regulate expression of HER2 [4] and contribute to the development of resistance to HER2-targeted therapies in breast cancer patients [5], we sought to establish if there was a prognostic relationship between JAM-A and HER2 in GE cancers.

In this study we report a significant relationship between JAM-A mRNA expression and survival in HER2-positive gastric cancer patients. However, due to the extremely heterogeneous expression of JAM-A protein in GE cancer tissue microarray (TMA) cores, no simple association between JAM-A and HER2 expression could be demonstrated in this reductionist model of study. This may reflect significant heterogeneity of HER2 protein expression within gastric tumors, which has been suggested to predict poor response to HER2-targeted therapy in patients [17]. However, the ability to capture the heterogeneity of JAM-A expression within a pilot set of full-face GE cancer tissue sections revealed correlations between JAM-A and HER2 expression that would have gone undetected using the TMA model. Specifically, utilizing a novel weighted-ranking scoring system for immunohistochemical expression of JAM-A in the larger tissue sections, significant correlations between the expression of JAM-A and HER2 were observed. Since immunohistochemistry (IHC) for protein markers is increasingly used to guide the choice of targeted therapies in cancer patients (for example, expression of the programmed death ligand-1 PD-L1 in selecting candidates for immunotherapy [18,19]); it is therefore timely and important for the cancer research community to acknowledge the influence of intra-tumor heterogeneity on biomarker outcome studies and to discuss reasonable frameworks to overcome potential bias.

## 2. Results

**Figure 1:**
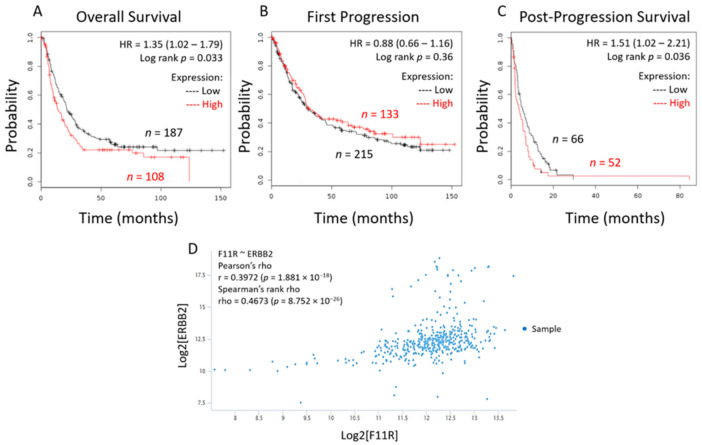
High JAM-A and HER2 expression predict worsened survival in gastric cancer patients. The Kaplan–Meier Plot platform (http://kmplot.com/analysis/ (accessed on 3 July 2019)) was used to generate survival curves in HER2-positive gastric cancer patient populations with high or low mRNA expression of JAM-A. Using F11R Jetset probes (excluding GSE62254 as recommended) in HER2-positive cases revealed that high mRNA expression of F11R (JAM-A) was associated with worsened overall survival (**A**) and post-progression survival (**C**), but not time to first progression (**B**). The Xenabrowser platform (https://xenabrowser.net/ (accessed on 28 August 2019)) was used to generate a scatter plot (**D**) showing correlations between JAM-A and HER2 mRNA expression in gastric cancer patients. Using the JAM-A and HER2 genes (named F11R and ERBB2, respectively) as variables in the TCGA Stomach Cancer cohort, a statistically significant moderately positive correlation was observed (**D**).

To begin testing the clinical significance of JAM-A expression in HER2-positive gastric cancer patient cases, we used an open-source online gene expression data resource (http://kmplot.com (accessed on 3 July 2019)) [20]. Analysis revealed that high expression of JAM-A mRNA in HER2-positive GE cancers carried a significantly greater risk of both poorer overall patient survival (*p* = 0.033; Figure 1A) and poorer survival in patients whose cancers progressed (*p* = 0.036; Figure 1C), although this was independent of the time to progression (Figure 1B). A second open-source online data resource (http://xenabrowser.net (accessed on 28 August 2019)) confirmed a moderate positive correlation between high JAM-A and high HER2 mRNA expression in a gastric cancer patient cohort (*r* = 0.4, *p* < 0.001; Figure 1D).

**Figure 2:**
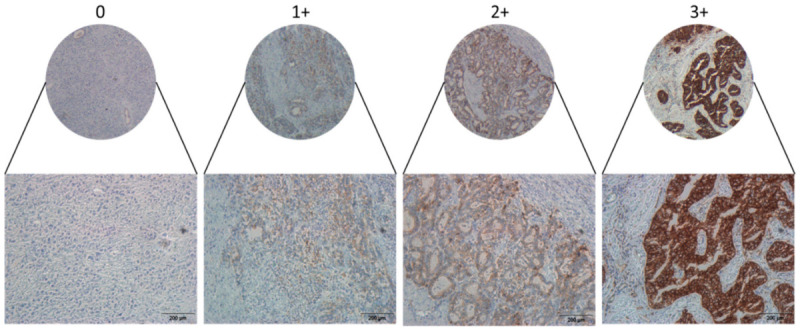
JAM-A immunohistochemical scoring in a GE cancer tissue microarray. JAM-A immunohistochemistry (IHC) was performed on 4 µm sections across a TMA of GE cancers totaling 174 primary tumor cases with 150 scorable cases (of which 43 had duplicate cores). Membranous JAM-A expression was scored 0, 1+, 2+, 3+ based on completeness and intensity of staining. Scale bar = 200 µm.

We therefore sought to validate these results at protein level, by immunohistochemically staining a 174-core GE cancer tissue microarray (TMA) [21] for JAM-A expression. In total, 150 cores were successfully scored for JAM-A membranous staining as described (Figure 2), with 45 primary tumors scored as having high (3+) JAM-A expression. In the event of a discordant score between matched duplicate patient cores, the higher score was adopted for the purposes of analysis. The relationship between JAM-A expression and clinicopathological features was then examined using χ^2^ crosstabulation and the asymptotic significance was calculated. Surprisingly, analysis revealed no significant correlation between JAM-A expression and tumor grade, proliferation (Ki67 staining), Lauren classification, differentiation, TNM-staging or HER2-positivity (Table 1). There was also no significant correlation between JAM-A expression and overall survival (Table 1). However, significant (*p* < 0.05) correlations were seen between JAM-A expression and primary tumor location, with high JAM-A levels being more frequent in esophageal than in stomach cancer cases.

JAM-A expression in a TMA with samples from 174 chemoradio-naïve primary tumors from GE cancer patients was stratified based on either high (3+) or low/moderate (1+, 2+) JAM-A expression based on intensity and completeness of JAM-A membranous staining. Statistical analysis was carried out using the χ^2^ test comparing JAM-A expression with clinicopathological parameters on SPSS version 24 software. Comparisons were considered significant at *p* < 0.05 (*); and all *p* values are shown in bold type. High JAM-A levels were significantly more likely in esophageal than stomach cancer cases.

**Figure 3:**
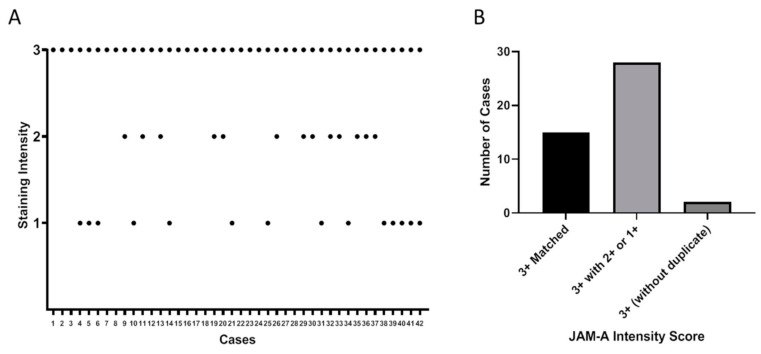
JAM-A protein expression is heterogeneous across duplicate cores. Duplicate cores assessed showed discordance within 43 individual cases (**A**,**B**), where at least one core scored 3+ for JAM-A protein expression intensity.

Given previously published correlations between high JAM-A expression, poor patient prognosis and HER2-positivity [4,6], these data were initially surprising. However, the discordance between scores for duplicate cores was noteworthy in that it highlighted significant intra-tumoral heterogeneity of JAM-A expression in this cancer tissue setting (Figure 3A). Specifically, out of the 45 JAM-A-high patient cases (of which 43 had duplicate cores); only 15/43 (35%) of duplicates had matching scores while 28/43 (65%) did not (Figure 3B).

Since intra- tumor heterogeneity may have a significant impact on tumor progression and patient prognosis [22], it was therefore necessary to consider alternative methods for assessing JAM-A expression which might take this into account.

**Figure 4:**
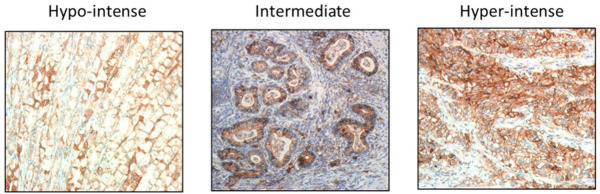
JAM-A expressional scoring in gastric full-face sections using a novel scoring algorithm. JAM-A immunohistochemistry (IHC) was performed on 4 µm sections across 25 primary GE cancer cases. Membranous JAM-A expression was scored 0, 1+, 2+, 3+ based on completeness and intensity of staining; with each score given a % value for each section. Each percentage scored 1+ was then multiplied by 0.25, each percentage scored 2+ was multiplied by 0.5 and each percentage scored 3+ was multiplied by 1. The scores were then totaled to give a cumulative JAM-A intensity score. Cumulative scores < 33% were denoted as hypo-intense, 33–66% were deemed intermediate and scores > 66% were assessed as being hyper-intense for JAM-A expression. Images were obtained at 20× magnification.

Accordingly, a separate immunohistochemical study using 25 full-face GE cancer patient tissue sections was undertaken to test whether JAM-A expression on larger tissue surfaces better predicted an association with the clinicopathological features of GE cancer patients. Fourteen of the 25 patient sections were full-face versions of cases represented in the TMA; with intra-tumoral heterogeneity being further illustrated by the fact that 9 of the 13 cases with duplicate TMA cores exhibited a discordant score between those duplicates (Figure S1). Even of the four cases that scored identically between duplicate cores, only two of them agreed with the majority score from their corresponding full-face section (Figure S1). High (3+) expression of JAM-A was observed in 23 out of 25 cases, but often only in small focal areas rather than reflecting the majority score of the section. To capture the relevance of this significant expressional heterogeneity within individual cases, a weighted ranking system was developed in which each section was assigned percentages for each score (0, 1+, 2+, 3+). Each percentage was then weighted, and the weighted totals used to assign an overall designation of hypo-intense, intermediate, or hyper-intense JAM-A expression (Figure 4 and Table 2). JAM-A expression was hyper-intense in 44% of cases, intermediate in 48% of cases and hypo-intense in 8% of cases.

**Figure 5:**
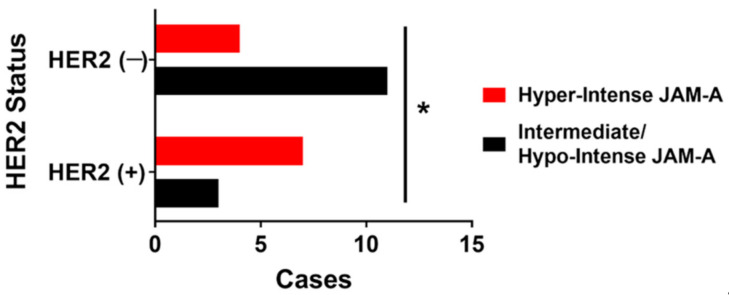
High JAM-A expression in full-face sections of GE cancer cases is significantly associated with HER2 positivity. Hypo-intense/intermediate or hyper-intense JAM-A expression in 25 full-face sections of primary GE tumors (moderately or poorly differentiated) was correlated with HER2 status, where HER2 was deemed positive with an IHC score ≥ 2+. Statistical analysis consisted of Fisher’s exact test using GraphPad Prism version 8.4.3 (GraphPad Software, San Diego, CA, USA); with comparisons considered significant at * *p* < 0.05.

There was no correlation between the cumulative JAM-A intensity score and T or N staging, differentiation status, Lauren classification or tumor location (Table 3). However, hyper-intense JAM-A expression correlated significantly with HER2-positivity; with 70% of HER2-positive cases having hyper-intense JAM-A expression and 73% of HER2-negative cases having only hypo-intense or intermediate JAM-A staining; (Figure 5; *p* < 0.05).

## 3. Discussion

The normal physiological role of JAM-A in tissues is that of cell-adhesion [23]. Recently, however, JAM-A overexpression has been highlighted as having a pathophysiological role in various cancers [24] and demonstrated to be a potential regulator of HER2 in HER2-positive breast cancers [4,5]. Since the clinical significance of JAM-A overexpression in GE cancer is currently controversial [12,13], this study therefore set out to test whether there was an expressional association between JAM-A and HER2 in HER2-overexpressing GE cancers.

Interrogation of two online databases revealed a significant relationship between JAM-A mRNA expression, HER2 status and patient survival. Surprisingly, however, analysis of JAM-A immunohistochemical staining in a 174-case GE tumor TMA did not reveal any significant correlations between JAM-A protein expression and clinicopathological features such as tumor grade, proliferation, Lauren classification, differentiation, TNM-staging or HER2-positivity. There was however a significant correlation between JAM-A expression and primary tumor location, with high JAM-A expression associating more frequently with tumors of esophageal than of gastric origin. These limited associations were unexpected in light of recent work supporting associations between JAM-A expression and key clinicopathological factors in some cancers [6,7,12]; and they naturally suggest the advisability of broadening any limited methodological study into future work with greater patient numbers, a wider representation of ages or cancer subtypes, and a capacity to mechanistically investigate any observations made.

However, the work illustrates an important aspect on the use of cancer TMAs for assessing proteins known to be heterogeneously expressed. Given the noted heterogeneity of JAM-A expression in this setting, and the novelty of understanding its relevance here, it is essential that consideration of intra-tumoral heterogeneity be factored into any initial discovery research. Full-face sections enabled the examination of larger areas and hence facilitated a greater appreciation of the intra-tumoral heterogeneity of JAM-A protein expression. Accordingly, a statistically significant association between JAM-A and HER2 expression was revealed in full-face sections when heterogeneity levels had been accounted for-an association that was not visible by TMA analysis alone. Importantly, the correlative trend between JAM-A and HER2 protein expression also recapitulated gene expression results from online gastric cancer datasets, suggesting a broader link between the two markers. It is interesting to note that significant intra-tumoral heterogeneity has already been observed at single cell level for HER2 gene expression in breast cancer patients [25]; and indications suggest that HER2 gene and protein expression is even more heterogeneous within gastric tumors [17]. It is likely that these features directly impact patient prognosis rather than being simply epi-phenomena; with evidence that intra-tumoral *HER2* gene copy number heterogeneity marks poor disease-free survival in breast cancer patients [25], and that high intra-tumoral heterogeneity of HER2 protein expression predicts poor progression-free survival and overall survival in gastric cancer patients [17]. 

The experimental findings described above which noted significant correlations between JAM-A/HER2 expression in full-face but not TMA sections were made possible by the development of a novel weighted ranking system which quantified cumulative JAM-A protein staining intensity across heterogeneous areas of a full-face section. The weighted scores were then combined into a cumulative JAM-A intensity score defined as either hypo-intense, intermediate, or hyper-intense. Once cumulative scores had been allocated, sections were re-examined to test whether the assessment had accurately captured JAM-A expression for each case. On reflection, cases had been accurately divided into sub-groups based on JAM-A staining using the novel weighted-ranking system. However, it is important to note that the full-face GE sections analyzed in this study all came from patients with poorly or moderately differentiated tumors, and amounted to a modest number of 25 cases. Thus, whether or not the weighted ranking system described herein will apply also to highly differentiated tumors is currently unknown. Since high JAM-A expression often correlates with poor differentiation status in cancers associated with other tissues [4,8,9,10,11], it is possible that the weighted ranking system may have limited applicability in well-differentiated GE tumors. However, since well-differentiated tumors pose a lower clinical risk and have a broader suite of treatment options, it is reasonable to argue that biomarker development is most urgent and relevant in the settings of poorly or moderately differentiated GE cancers. Regardless, an attempt to describe the inherent heterogeneity of such cases is essential in validation studies; not just to accurately capture information about a given protein in specific tissues, but also to translate its importance into the setting of intra-tumoral heterogeneity and the role it may play in disease pathogenesis [22]. Furthermore, it will be important in future disease models to validate the extent and consequences of intra-tumoral heterogeneity for disease progression or sensitivity to treatment. For example, in a chick embryo xenograft model of GE cancer, we observed highly variable intra-xenograft patterns of JAM-A protein expression (Figure S2). Specifically, there was highly heterogeneous expression of JAM-A both in control xenografts treated with a non-targeting siRNA (siN) or xenografts in which JAM-A had been transiently silenced using specific siRNA (siJp). Despite a low transfection efficiency, the heterogeneity of JAM-A expression quite reasonably recapitulated the intra-tumoral heterogeneity observed in real patient cases. Furthermore, it was encouraging that JAM-silenced xenografts scored lower for JAM-A expression than control xenografts; even if this was insufficient to translate into either gross (Figure S3) or microscopic (Figure S4) reductions in xenograft tumor size or invasive characteristics over a short experimental time course.

In a wider context, there are several methods for quantifying the presence of important markers in cancer tissues depending on the type of protein under examination. However, the clinical utility of these markers depends on several factors, not least the ease and reproducibility of assessment. For example, the proliferation marker Ki67 is frequently measured in the binary sense as either negative or positive (recently reviewed in [26]), although some pathologists examine a random area of tissue and assess the percentage positivity. Semi-quantitation of prognostically and therapeutically useful markers such as HER2 is also common, with HER2 itself being scored on the intensity of membranous staining (strong, complete = 3+, weak to moderate complete = 2+, weak, incomplete = 1+ and no evident staining = 0) [27,28]. However, given its sometimes subjective nature, discordant IHC scoring can lead to varying cut-off thresholds, as well as varying assay methods for assessing the expression of clinically important markers such as PD-L1 [29]. The potential clinical ramifications of such uncertainty are well illustrated by PD-L1, since reporting of its expression levels currently influences patient selection for immunotherapies. Therefore, the need for unification of assessment for proteins like PD-L1 highlights a timely and important issue in histopathology.

## 4. Materials and Methods

### 4.1. Tissue Microarray Staining

A TMA with samples from a previously described cohort of 174 primary chemoradio-naïve GE cancers with known HER2 status and other clinicopathological features [21] was stained for JAM-A expression (antibody #H00050848-M01, 0.4 μg/mL, Abnova, Taiwan). Membranous JAM-A expression was subsequently quantified by two independent authors (CER and KMS) using a 0, 1+, 2+, 3+ semi-quantitative scoring system as described in the following methods. All images were obtained using an Olympus CKx41 microscope with Cell B imaging software at 20× magnification. Analysis was conducted using SPSS version 24 software (IBM, Armonk, NY, USA). 

### 4.2. Full-Face Gastric Cancer Tissue Staining

GE cancer tissue sections (4 μm) from *n* = 25 patients were stained for JAM-A (Abnova cat #H00050848-M01) using a Leica Bond III automated IHC stainer. Semi-quantitative scoring of JAM-A membranous staining was completed blindly by two or three authors (CER, KMS, EWK). Analysis was accomplished using a weighted ranking system, allocating 0.25 for every % on each section scored as 1+, 0.5 for every % scored as 2+ and 1 given for every % scored as 3+, leaving multiple intensities for each section. Based on this, an overall cumulative score was then calculated per tissue section and ranked as either hypo-intense (<33%), intermediate (33–66%) or hyper-intense (>66%) for JAM-A staining based on the overall score. All images were obtained using an Olympus CKx41 microscope with Cell B imaging software at 20× magnification. Analysis was conducted using SPSS version 24 software (IBM, Armonk, NY, USA).

## 5. Conclusions

In conclusion, this report has presented a novel weighted ranking assessment method for assessing the levels of heterogeneously expressed biomarker proteins. Although JAM-A expression in GE cancer tissue was the focus of this study, a similar weighted ranking assessment has potential applications for other proteins like PD-L1, for whom heterogeneity of expression is an ongoing issue [30]. A potential limitation of this approach is in its laboriousness, which might hamper its practical utility in clinical diagnostic laboratories. However, given the rapid pace of development of software algorithms to quantify IHC protein expression, it is reasonable to speculate that this approach could be automated to minimize the labor-intensiveness of semi-quantitatively scoring tissues in this manner. Overall, we believe this method for assessment of heterogeneously expressed prognostic and/or predictive biomarkers could lead to improved patient stratification for targeted therapies under development. Given that JAM-A has been highlighted as a potential biomarker for worsened prognosis in some cancers [6,12], as well as a potential novel indicator of resistance to HER2-targeted therapies in breast cancer patients [5], it is important that its relevance in this setting is fully elucidated and its intra-tumoral heterogeneity considered as a potential parameter for patient outcomes and response prediction.

## Figures and Tables

**Table 1:** Associations between JAM-A expression and clinicopathological features.

Variable	N	Low/Moderate	High	*p*-Value
**Sex**	150			**0.596**
Female		23	9	
Male		79	39	
**Overall Survival**	150			**0.735**
Living		25	13	
Deceased		77	35	
**T-Stage**	150			**0.843**
1		9	5	
2		18	11	
3		57	25	
4		18	7	
**N-Stage**	150			**0.981**
0		29	15	
1		20	9	
2		28	12	
3	150	25	12	
**M-Stage**				**0.107**
0		89	47	
1		13	1	
**Differentiation**	150			**0.664**
High		5	1	
Moderate		32	17	
Low		65	30	
**Lauren Classification**	150			**0.359**
Intestinal		72	39	
Diffuse		25	7	
Mixed		5	2	
**Location**	150			* 0.049
Esophagus + GE junction		62	37	
Stomach		40	11	
**Ki67**	149			**0.545**
0–1%		2	1	
2–10%		14	4	
11–20%		17	11	
21–50%		29	18	
>50%		39	14	
**HER2 Positivity**	96			**0.396**
Negative		54	24	
Positive		9	4	
Unspecified		2	3	
HER2 Positivity				

**Table 2:** Weighted ranking system for immunohistochemical scoring of JAM-A on full-face GE cancer tissue sections.

GE Full-Face Section Overall Score Calculation									
Case #	% of Score			Grade Calculation			Accumulated Grade Score	JAM-A Staining Intensity	HER2
	**1+**	**2+**	**3+**	**1+**	**2+**	**3+**			
1	20	50	30	5	25	30	60	Intermediate	−
2	-	40	60	-	20	60	80	Hyper-intense	+
3	5	55	40	1.25	27.5	40	68.75	Hyper-intense	+
4	33	33	33	8.25	16.5	33	57.75	Intermediate	−
5	5	55	40	1.25	27.5	40	68.75	Hyper-intense	+
6	30	40	30	7.5	20	30	57.5	Intermediate	−
7	10	60	30	2.5	30	30	62.5	Intermediate	+
8	10	20	70	2.5	10	70	82.5	Hyper-intense	+
9	75	25	-	18.75	12.5	-	31.25	Hypo-intense	−
10	10	40	50	2.5	20	50	72.5	Hyper-intense	+
11	30	30	40	7.5	15	40	62.5	Intermediate	−
12	10	20	70	2.5	10	70	82.5	Hyper-intense	−
13	45	50	5	11.25	25	5	41.25	Intermediate	−
14	80	20	-	20	10	-	30	Hypo-intense	−
15	-	20	80	-	10	80	90	Hyper-intense	−
16	-	-	100	-	-	100	100	Hyper-intense	+
17	30	60	10	7.5	30	10	4705	Intermediate	−
18	10	60	30	2.5	30	30	62.5	Intermediate	+
19	-	25	75	-	12.5	75	87.5	Hyper-intense	+
20	5	50	45	1.25	25	45	71.25	Hyper-intense	−
21	25	65	10	6.25	32.5	10	48.75	Intermediate	−
22	-	10	90	-	5	90	95	Hyper-intense	−
23	10	60	30	2.5	30	30	62.5	Intermediate	+
24	40	45	15	10	22.5	15	47.5	Intermediate	−
25	30	30	40	7.5	15	40	62.5	Intermediate	−
Grade 1+ = each % is worth 0.25				Hypo-intense = <33					
Grade 2+ = each % is worth 0.5				Intermediate = 33–66					
Grade 3+ = each % is worth 1				Hyper-intense = >66					

A novel weighted ranking system was developed in order to stratify cases based on heterogeneous JAM-A expression in *n* = 25 GE cancer cases. JAM-A expression evaluation revealed 11 cases to be hyper-intense, 12 cases to be intermediate and 2 cases as hypo-intense.

**Table 3:** Hyper-intense JAM-A expression significantly correlates with HER2 positivity in GE cancer full-face sections.

Variable	N	Hypo-Intense/Intermediate	Hyper-Intense	*p*-Value
**Overall Survival**	14			**0.825**
Living		1	1	
Deceased		7	5	
**T-Stage**	25			**0.536**
1		0	1	
2		1	1	
3		10	8	
4		3	1	
**N-Stage**	25			**0.45**
0		3	5	
1		4	2	
2		4	1	
3		3	3	
**Differentiation**	25			**0.42**
Moderately		2	3	
Poorly		12	8	
**Lauren Classification**	25			**0.692**
Intestinal		8	7	
Diffuse		1	0	
Mixed		5	3	
**Location**	25			**0.678**
Esophagus + Cardia		10	7	
Stomach		4	4	
**HER2 Positivity**	25			* 0.032
Negative		11	4	
Positive		3	7	

Full-face sections of GE cancer patient tissue (*n* = 25; all either moderately or poorly differentiated) were stratified based on either hyper-intense or intermediate/hypo-intense JAM-A expression based on intensity and completeness of JAM-A membranous staining. Statistical analysis was carried out using χ^2^ test comparing JAM-A expression with clinicopathological parameters on SPSS version 24 software. Comparisons were considered significant at *p* < 0.05 (*); and all *p* values are shown in bold type. JAM-A Expression: Hypo-intense/intermediate = cumulative intensity score ≤66; Hyper intense = cumulative intensity score >66; HER2 Positive = IHC ≥2+.

## Data Availability

The data presented in this study are available on request from the corresponding author.

## Supplementary Materials

The following are available online at https://www.mdpi.com/2072-6694/13/6/1286/s1, Figure S1: GE patient tumors exhibit significant intra-tumoral heterogeneity of JAM-A protein expression, Figure S2: JAM-A is heterogeneously expressed in chick embryo tumor xenografts of human GE cancer, Figure S3: JAM-A mediated silencing did not alter grossly-visible GE tumor development in a chick embryo xenograft model, Figure S4: JAM-A silencing did not microscopically alter tumor formation or invasion in a chick embryo xenograft model.
