# Supplementary material for: Development of a Novel Weighted Ranking Method for Immunohistochemical Quantification of a Heterogeneously Expressed Protein in Gastro-Esophageal Cancers
Source: Cancers (Basel). 2021 Mar 13;13(6):1286. doi: 10.3390/cancers13061286 (PMC7998246; doi:10.3390/cancers13061286)
Supplement: Supplementary file 1 [file cancers-13-01286-s001.pdf]

Supplementary Materials

Development of a Novel Weighted Ranking Method for Immunohistochemical Quantification of a Heterogeneously Expressed Protein in Gastro-Esophageal Cancers

Cathy E. Richards, Katherine M. Sheehan, Elaine W. Kay, Charlotta Hedner, David Borg, Joanna Fay, Anthony O'Grady, Arnold D. K. Hill, Karin Jirström and Ann M. Hopkins

	Full-Face Section				TMA		
**Case #**	**1+ (%)**	**2+ (%)**	**3+ (%)**	**JAM-A Intensity Staining**	**Core 1**	**Core 2**	
12	10	20	70	Hyper-intense	3+	3+	
13	45	50	5	Intermediate	1+	2+	
14	80	20	-	Hypo-intense	1+	1+	
15	-	20	80	Hyper-intense	3+	1+	
16	-	-	100	Hyper-intense	2+	3+	
17	30	60	10	Intermediate	3+	1+	
18	10	60	30	Intermediate	3+	1+	
19	-	25	75	Hyper-intense	3+	1+	
20	5	50	45	Hyper-intense	1+	3+	
21	25	65	10	Intermediate	1+	-	
22	-	10	90	Hyper-intense	2+	2+	
23	10	60	30	Intermediate	1+	2+	
24	40	45	15	Intermediate	1+	1+	
25	30	30	40	Intermediate	1+	2+	

	Matched core scores
	Discordant core scores
	Duplicate cores unavailable

Figure S1. GE patient tumors exhibit significant intra-tumoral heterogeneity of JAM-A protein expression. JAM-A protein expression was immunohistochemically stained in full-face sections and TMA cores from $n = 14$ GE cancer patients. Membranous JAM-A expression in TMA cores was semi-quantitatively scored by two blinded observers as 0, 1+, 2+, 3+ based on completeness and intensity of staining. In full-face sections, the relative percentages of areas scoring 0, 1+, 2+, 3 staining were recorded; with each score given a weighting. Cumulative scores of <33% were denoted as hypo-intense, 33–66% were deemed intermediate and scores >66% were defined as hyper-intense for JAM-A expression. TMA cores were considered "matched" (blue boxes) if duplicate cores scored the same, and "discordant" (red boxes) if duplicate cores did not score the same. Of the 13 cases that had available duplicate TMA cores, only 4 scores matched and 9 were discordant. Of the 4 TMA cases with matched scores, only 2 agreed with the majority score from their corresponding full-face section.

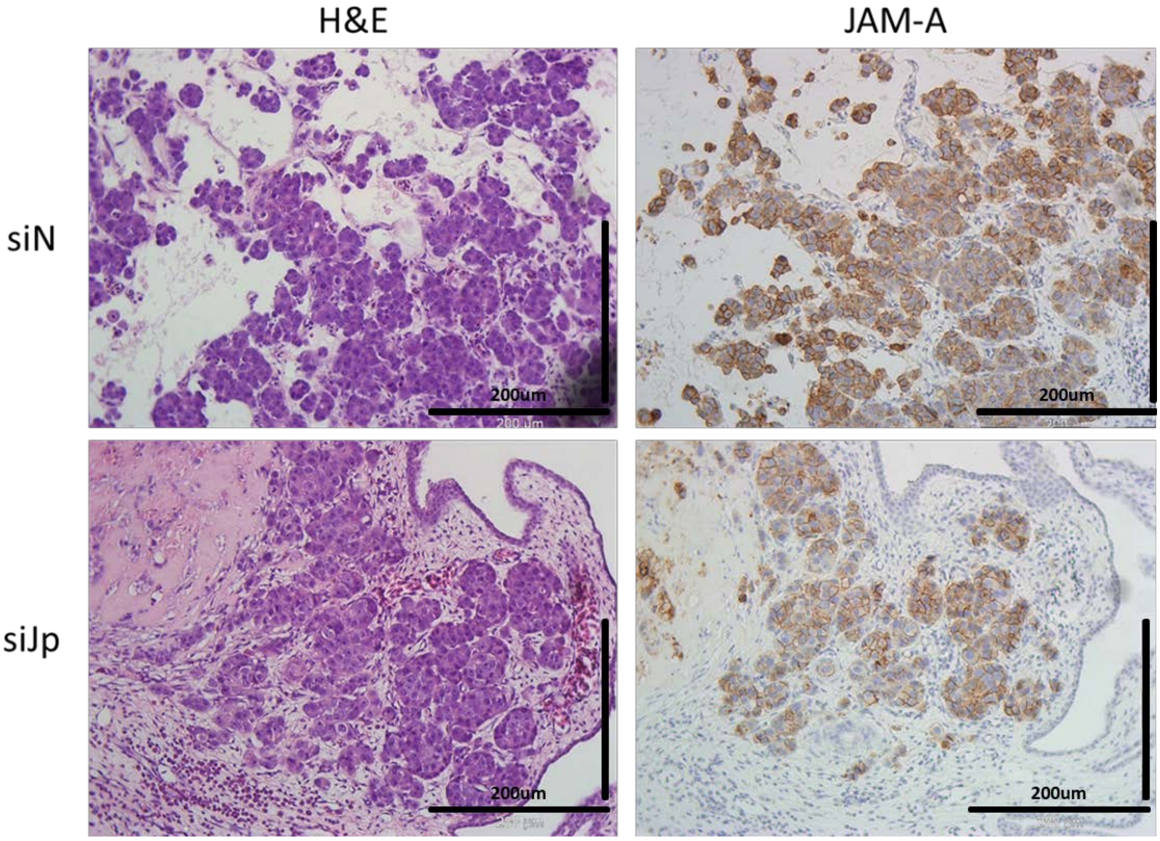

Figure S2. JAM-A is heterogeneously expressed in chick embryo tumor xenografts of human GE cancer. 2 × 10^6 ESO26 GE cancer cells were implanted onto the chorioallantoic membrane (CAM) of fertilized chicken eggs on day 8 of embryonic development; having been silenced 24 h earlier with 25 nM siRNA against JAM-A (siJp) or non-targeting control (siNeg). Xenografts were re-transfected with siRNA in situ on days 10 and 13 of embryonic development. On day 14 of embryonic development, xenografts and their surrounding CAM were excised, formalin-fixed and immunohistochemically stained for JAM-A expression. Significant heterogeneity of JAM-A expression was noted in both JAM-A-silenced and control conditions. Scale bar = 200 μm.

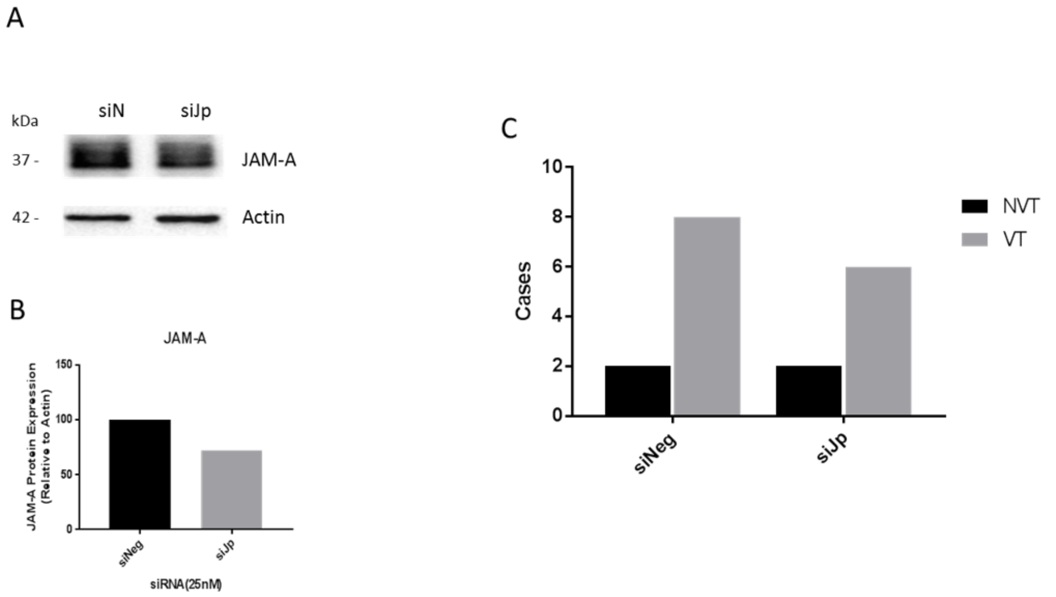

Figure S3. JAM-A mediated silencing did not alter grossly-visible GE tumor development in a chick embryo xenograft model. ESO26 GE cancer cells at 80% confluence were transfected with either 25 nM control siRNA (siNeg) or siRNA against JAM-A (siJp) and 2 × 10^6 cells implanted 24 h later onto the chorioallantoic membrane (CAM) of fertilized chicken eggs on day 8 of embryonic development. Some cells were reserved for protein extraction and Western blotting to confirm that JAM-A silencing had started by 24 h (**A**), with individual biological replicates densitometrically analyzed relative to a loading control (Actin). (**B**). Xenograft tumors were allowed to grow for 6 days on the CAM (with re-silencing performed *in situ* on days 10 and 13 of embryonic development after implantation on day 8), and examined for grossly-visible tumors. Xenograft tumors in siNeg versus siJp conditions were recorded as "visible tumor" (VT) or "no visible tumor" (NVT), and statistically compared by two-tailed Fisher's exact test (**C**). No significant differences were observed between treatment groups.

Supplemental Figure S3A	Original JAM-A Protein	Original Actin Protein
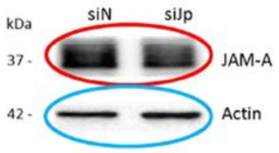	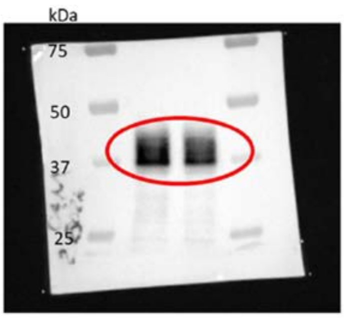	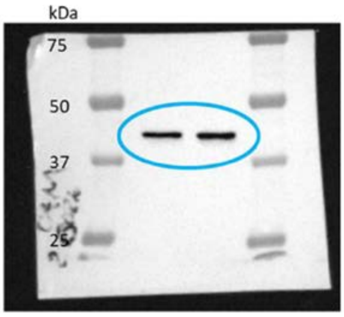

Uncropped blots for Figure S3A.

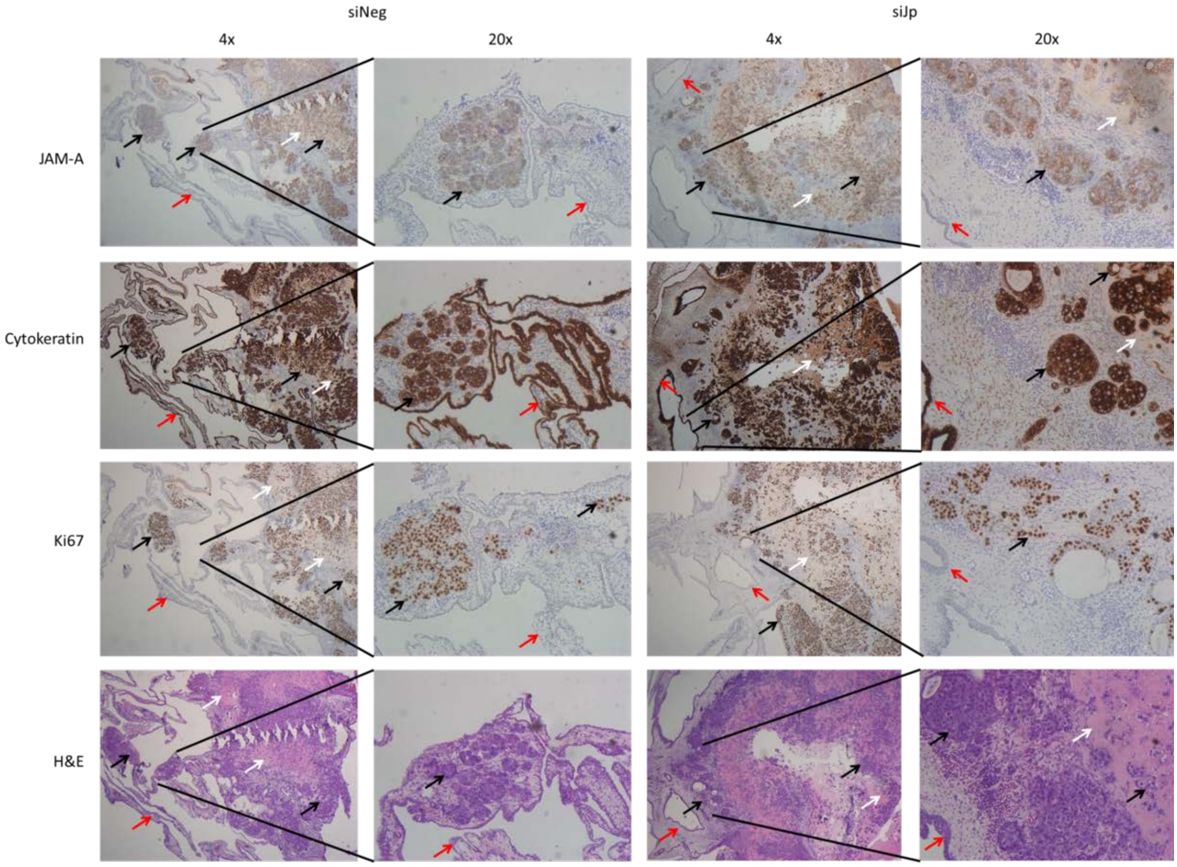

Figure S4. JAM-A silencing did not microscopically alter tumor formation or invasion in a chick embryo xenograft model. 2 × 10^6 ESO26 cells were implanted onto the chorioallantoic membrane (CAM) of fertilized chicken eggs on day 8 of embryonic development; having been silenced 24 h earlier with 25 nM siRNA against JAM-A (siJp) or non-targeting control (siNeg). Xenografts were re-transfected in situ on days 10 and 13. On day 14 of embryonic development, xenografts with their surrounding CAM were excised, formalin-fixed and stained for JAM-A, pan-cytokeratin, Ki67 and hematoxylin/eosin (H&E). Areas of tumor, Matrigel and CAM are denoted by respectively black, white and red arrows. All images were obtained using an Olympus CKx41 microscope with Cell B imaging software at 20× magnification.

© 2021 by the authors. Licensee MDPI, Basel, Switzerland. This article is an open access article distributed under the terms and conditions of the Creative Commons Attribution (CC BY) license (http://creativecommons.org/licenses/by/4.0/).
